# Diagnostic performances of the fluorescent spot test for G6PD deficiency in newborns along the Thailand-Myanmar border: A cohort study

**DOI:** 10.12688/wellcomeopenres.13373.1

**Published:** 2018-01-02

**Authors:** Laurence Thielemans, Gornpan Gornsawun, Borimas Hanboonkunupakarn, Moo Kho Paw, Pen Porn, Paw Khu Moo, Bart Van Overmeire, Stephane Proux, François Nosten, Rose McGready, Verena I. Carrara, Germana Bancone

**Affiliations:** 1 Shoklo Malaria Research Unit, Mahidol-Oxford Tropical Medicine Research Unit, Faculty of Tropical Medicine, Mahidol University, Mae Sot, 63110, Thailand; 2 Neonatology-Pediatrics, Cliniques Universitaires de Bruxelles - Hôpital Erasme, Université Libre de Bruxelles, Bruxelles, 1070, Belgium; 3 Mahidol-Oxford Tropical Medicine Research Unit (MORU), Faculty of Tropical Medicine, Mahidol University, Bangkok, 10400, Thailand; 4 Centre for Tropical Medicine and Global Health, Nuffield Department of Medicine, University of Oxford, Oxford, OX3 7BN, UK

**Keywords:** G6PD deficiency, Fluorescent spot test, Umbilical cord blood, quality control, neonatal screening

## Abstract

**Background: **Glucose-6-phosphate dehydrogenase (G6PD) deficiency is an inherited enzymatic disorder associated with severe neonatal hyperbilirubinemia and acute haemolysis after exposure to certain drugs or infections. The disorder can be diagnosed phenotypically with a fluorescent spot test (FST), which is a simple test that requires training and basic laboratory equipment. This study aimed to assess the diagnostic performances of the FST used on umbilical cord blood by locally-trained staff and to compare test results of the neonates at birth with the results after one month of age.

**Methods**: We conducted a cohort study on newborns at the Shoklo Malaria Research Unit, along the Thai-Myanmar border between January 2015 and May 2016. The FST was performed at birth on the umbilical cord blood by locally-trained staff and quality controlled by specialised technicians at the central laboratory. The FST was repeated after one month of age. Genotyping for common local G6PD mutations was carried out for all discrepant results.

**Results: **FST was performed on 1521 umbilical cord blood samples. Quality control and genotyping revealed 10 misdiagnoses. After quality control, 10.7% of the males (84/786) and 1.2% of the females (9/735) were phenotypically G6PD deficient at birth. The FST repeated at one month of age or later diagnosed 8 additional G6PD deficient infants who were phenotypically normal at birth.

**Conclusions**: This study shows the short-comings of the G6PD FST in neonatal routine screening and highlights the importance of training and quality control. A more conservative interpretation of the FST in male newborns could increase the diagnostic performances. Quantitative point-of-care tests might show higher sensitivity and specificity for diagnosis of G6PD deficiency on umbilical cord blood and should be investigated.

## Introduction

Glucose-6-phosphate dehydrogenase (G6PD) deficiency is the most prevalent X-linked enzymatic deficiency worldwide ^1^. Full enzymatic deficiency is observed in mutated hemizygous males and mutated homozygous females ^2^. Blood from heterozygous females contains both normal and deficient red blood cells in variable proportions, with a large distribution of enzymatic activities at population level. In populations with a high prevalence of G6PD deficiency, enzymatic activities have a bimodal distribution in adult males and a continuous distribution in adult females. Although most individuals with G6PD deficiency are asymptomatic, the genetic condition can cause acute haemolysis after exposure to fava beans, certain drugs, chemicals, or infections. In neonates, G6PD deficiency increases the risk of sepsis ^3^, severe neonatal hyperbilirubinemia, and kernicterus ^4^. G6PD deficiency is a risk factor for neonatal hyperbilirubinemia, in mutated hemizygous males ^5^ and mutated homozygous and heterozygous females ^6^, even without exposure to known haemolytic agents ^7^. Early recognition of G6PD deficiency in newborns, prompting the implementation of useful prevention strategies such as targeted follow-up, counselling on trigger avoidance, promotion of breast milk intake, and the use of effective phototherapy, can help prevent kernicterus ^8^. Routine G6PD deficiency screening in newborns is recommended by the World Health Organization if the prevalence of G6PD deficiency reaches 3–5% among males ^9^.

The G6PD fluorescent spot test (FST) ^10^ is an inexpensive and reliable qualitative phenotypic test ^11, 12^; however it requires a cold chain for reagents and a basic training for the users, which limits its use in the field. The FST has a sensitivity and specificity above 95% for the diagnosis of G6PD deficiency in adults with less than 30% enzymatic activity ^12– 14^. With this threshold, the test correctly diagnoses mutated hemizygous males, mutated homozygous females and G6PD heterozygous females with low enzymatic activity. Subjects with intermediate to normal enzymatic activity are diagnosed as G6PD normal by this method.

The highest prevalence of G6PD deficiency is found in malaria endemic regions ^1, 15^. In the population attending Shoklo Malaria Research Unit (SMRU) clinics, the prevalence of G6PD deficiency is 13.7% in adult males ^16^ and 2–4% in adult females ^17^ and neonatal hyperbilirubinemia is common ^18^. Routine neonatal screening for G6PD deficiency is therefore recommended in this setting ^9^.

Umbilical cord blood collection is easy, can spare the neonate unnecessary pain, and results of cord blood G6PD FST are comparable to that of peripheral blood drawn in the first week of life in a recent study ^19^. However, there is evidence suggesting that the diagnosis performed at birth should be confirmed later in life, because of a potentially higher G6PD activity in neonates compared to adults ^20– 23^.

As part of a cohort study on neonatal hyperbilirubinemia in this population, we carried out an analysis with two objectives: to assess the diagnostic performance of the FST used by locally trained staff on umbilical cord blood, and to compare test results at birth and after one month of age.

## Methods

The study was conducted at the SMRU, located along the Thailand-Myanmar border in the north-western province of Tak, Thailand. The population attending SMRU clinics is composed of migrants and refugees. The staff of SMRU clinics consists of locally trained medics, midwives, nurses, health workers and laboratory technicians. The field clinics including the laboratories have stable electricity and are equipped with basic equipment and refrigerators.

Newborns enrolled in a cohort study between January 2015 and May 2016 ^24^ who were born after 28 weeks of gestational age and had a G6PD FST performed on umbilical cord blood were included in the analysis. The FST was performed using five microliters of blood that were mixed with 100 μl of reagents (R&D Diagnostic, Greece), incubated for 10 minutes at room temperature, spotted on filter paper and air dried. The spot tests were then visualised under UV light by a locally trained laboratory technician. Spot tests showing intermediate to normal fluorescence were classified as G6PD normal, while those showing no fluorescence were classified as G6PD deficient. Spot tests were transported within 12 hours to the central haematology laboratory in Mae Sot where they were re-examined by qualified laboratory technicians for quality control (QC). Data were analysed using SPSS (IBM SPSS Statistics 23, IBM Corporation). Sensitivity, specificity, negative predictive value (NPV), and positive predictive value (PPV) were calculated to evaluate the concordance between the FST performed in the clinics and the QC. The QC results were used to calculate the prevalence of G6PD phenotype at birth.

Neonates were followed up and after one month of age a capillary blood sample was tested with the FST by a locally trained laboratory technician. Samples with discrepant QC results at birth, and with different results at birth versus after one month of age were genotyped using established PCR-RFLP protocols for Mahidol, Chinese-4, Canton, Viangchan, Mediterranean and Kaiping mutations ^25^.

### Ethical statement

The study was approved by the Ethic Committees of the University of Oxford, UK (OXTREC 41-144) and the Faculty of Tropical Medicine, Mahidol University, Thailand (TMEC 14-012). The Tak Community Advisory Board, consisting of members of the local community, also revised and approved the study (TCAB-08-13).

The study was explained in the preferred local language by a trained counsellor during pregnancy and the mothers who agreed in participating signed an informed consent.

## Results

### G6PD testing on umbilical cord blood

FST on umbilical cord blood was performed in 1521 newborns; 51.7% (n=786) were males and 4.5% (n=68) were born before 37 weeks of gestation. Results of QC performed in the central laboratory were available for all tests. The results are shown in Table 1. Among the 1434 tests interpreted as normal at the clinic, 0.6% (6 males and 2 females) were re-classified as deficient by the QC. Among the 87 tests interpreted as deficient at the clinic, 2.3% (one male and one female) were re-classified as normal by the QC. Genotyping for the 10 discrepant results supported the interpretation of the QC. Proportions of errors in the interpretation of the test were similar across clinical sites. The calculated sensitivity, specificity, PPV and NPV for the FST at the clinics were 91.4% (95 % CI: 91.4- 91.4), 99.9% (95 % CI: 99.9- 99.9), 97.7% (95 % CI: 96.9-98.5) and 99.4% (95 % CI: 99.8-100.0) respectively.

**Table 1.** Results of the fluorescent spot test performed at the clinics and in the central laboratory (quality control).

		Quality control	
		Deficient	Normal
**Clinics**	Deficient	85	2 *
	Normal	8 **	1426
	Total	93	1428

*1 wild type male, 1 wild type female **6 hemizygous Mahidol males, 1 heterozygous Mahidol female and 1 homozygous Mahidol female

Overall, 10.7% of the males (84/786) and 1.2% of the females (9/735) were diagnosed with a G6PD deficient phenotype at birth.

### Repeated G6PD testing on infant after one month of age

**Figure 1.**
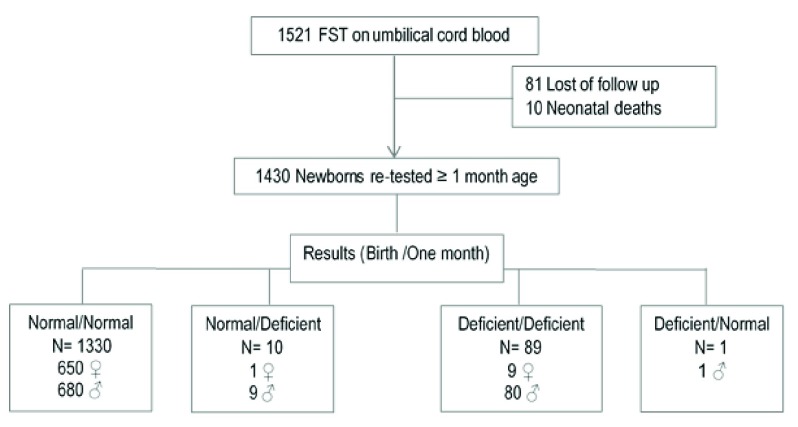
The number of patients analyzed at birth and after one month of age.

The FST was repeated at least one month after birth in 1430/1521 neonates ( Figure 1) and results were compared with those of the QC at birth ( Table 2).

**Table 2.** Results of the fluorescent spot test performed at birth and repeated after 1 month of age.

		FST ≥ 1 month of age	
		Deficient	Normal
**FST birth**	Deficient	89	1 *
	Normal	10 **	1330
	Total	99	1331

*Mahidol hemizygous male **7 Mahidol hemizygous male, 1 Canton hemizygous male, 1 wild type male, 1 wild type female

Among the 90 G6PD deficient infants at birth, one male had an FST performed after one month of age interpreted as normal. However, the genotyping showed that he was hemizygous for Mahidol variant, confirming the diagnosis of G6PD deficiency done at birth.

Among the 1340 G6PD normal newborns, 10 (9 males and one female) were diagnosed as deficient by FST when re-tested after one month of age. Genotyping confirmed that, among 8 males, 7 were hemizygous for Mahidol variant and one for Canton variant. The remaining two (one male and one female) had a wild type genotype for the tested mutations; we could not confirm whether they were carriers of uncommon G6PD mutations or the FST at follow-up was mis-interpreted *.* Overall, we estimated that at least 8 of the 99 deficient infants (8.1%) tested after one month of age were misdiagnosed as G6PD normal at birth.

## Discussion

The fluorescent spot test has been performed in the SMRU clinics for almost 10 years, showing reliable results in adult patients ^16^. It has also been used for diagnosis of G6PD deficiency in jaundiced neonates ^18^, but an assessment of diagnostic performance in routine testing of newborns has never been done until the current study. The results show that among 1521 newborns tested for G6PD deficiency, 0.6% were misclassified by the locally-trained clinical staff. Importantly, 8 of them, including 6 hemizygous males, were diagnosed as normal but the QC and genotyping showed they were indeed deficient, highlighting that the misclassification failed to identify 8.6% (8/93, Table 1) of G6PD deficient newborns, who would be at risk of developing hyperbilirubinemia ^9^.

Furthermore, when the FST was repeated in infants after one month of age, 10 infants diagnosed as normal at birth had a deficient FST and 8 of them were confirmed to be deficient by genotyping. The one infant who had a deficient FST at birth and a normal result at one month was confirmed as a mutated hemizygous male, suggesting that the result at one month follow up was a classification error.

Failure to diagnose G6PD deficiency at birth has been observed before in other settings ^22, 26, 27^. Blood samples from newborns have higher G6PD activity than adults ^20, 21^; since G6PD activity is higher in immature blood cells compared to mature erythrocytes ^28^, higher activity in neonatal blood could be explained by the higher number of reticulocytes ^29^ and the higher proportion of immature reticulocytes in umbilical cord blood ^30^. The FST is a qualitative test and only allows a reliable diagnosis for binary results whereby samples with intermediate fluorescence are assigned a normal phenotype. When the enzymatic activity of a sample is close to or slightly over the 30% threshold, as might be the case in deficient samples with reticulocytosis, the subjective interpretation of the test can become very difficult. In this study, we hypothesise that the experienced laboratory technicians gave a more conservative interpretation of the tests performed at birth than the locally-trained staff.

Our results highlight the importance of regular training for laboratory technicians and routine quality controls on test procedure and interpretation to assure stable performances of the FST; furthermore they indicate a specific short-coming of the use of a qualitative test for G6PD screening on umbilical cord blood samples. We suggest that the training of laboratory technicians focuses in particular on male newborns who have an FST with intermediate fluorescence. These neonates should be considered G6PD deficient because they are likely mutated hemizygous. In female newborns with an intermediate fluorescence FST, a systematic more conservative interpretation of tests would instead increase the number of false deficient results. The lower test specificity would likely increase health care workers workload and would create unnecessary stress and confusion in families. However, caretakers should be aware of the limited reliability of the test in newborns and should provide routine surveillance of neonates, parental education about exposure to triggers of haemolysis and signs of jaundice before discharge, regardless of the neonate’s G6PD diagnosis ^31^. In settings where the G6PD neonatal screening is carried out, re-testing at an older age is rarely done. G6PD status should be re-assessed when the child is at least 6 months old (when steady-state G6PD activity has reached the level seen in adulthood ^20^) if oxidant treatments need to be administered. This is especially important in some areas such as malaria endemic areas where a long course of primaquine is needed for *Plasmodium vivax* radical cure. 

In conclusion, the results of this study show that the FST does not perform well in newborns, suggesting that a quantitative G6PD test would be more sensitive in diagnosing neonates at risk of hyperbilirubinemia ^6, 21, 29, 32, 33^. Laboratory-based G6PD quantitative tests are expensive and require skilled technicians and well-equipped laboratories. However, point-of-care quantitative tests for G6PD have been developed in the past few years ^34– 36^. Upon validation, they should be available in resource-limited settings for the screening of G6PD deficiency in newborns.

## Data availability

Due to ethical and security considerations, the data that supports the findings in this study can be accessed only through the Data Access Committee at Mahidol Oxford Tropical Medicine Research Unit (MORU). The data sharing policy can be found here: http://www.tropmedres.ac/data-sharing. The application form for datasets under the custodianship of MORU Tropical Network can be found in Supplementary File 1.

## Supplementary material

Supplementary File 1: Application form for datasets.
