# Supplementary material for: Diagnostic performances of the fluorescent spot test for G6PD deficiency in newborns along the Thailand-Myanmar border: A cohort study
Source: Wellcome Open Res. 2018 Jan 2;3:1. [Version 1] doi: 10.12688/wellcomeopenres.13373.1 (PMC5829521; doi:10.12688/wellcomeopenres.13373.1)
Supplement: Peer review history

# Peer review: Referee response for version 1

**DOI:** 10.21956/wellcomeopenres.14520.r30456

**Published:** 2018-06-04

**Authors:** Tina M Slusher, Troy Lund

**Affiliations:** 1 University of Minnesota, Minneapolis, MN, USA; 2 Department of Pediatrics, University of Minnesota, Minneapolis, MN, USA; 3 Hennipen Health Care PICU Minneapolis, Minnesota, MN, USA

This paper accurately points out the advantages and disadvantages of the FST for detecting G6PD deficiency in neonates and adds to the body of literature out there regarding its use.  I however feel the authors overstate the limitations in the discussion when they say in the discussion that “the FST does not perform well”.  The FST is done almost exclusively in LMICs where quantitative tests are not routinely available and if available are often too expensive to use outside of a research study.

 1. Compared to the alternative which is usually no G6PD test having a false negative result of 0.6% is quite acceptable and still allows appropriate counseling and treatment of the majority of the neonates. 

 2. The do not provide the data to support their statement that that males with intermediate fluorescence be considered.  It would be better if they would supply this data. 

 3.The improved results in the central laboratory highlight the need for ongoing education, supervision and oversight needed when having a test run by non-laboratory personnel but again don’t negate the benefits of having the FST available for routine use. 3. Finally, no one would argue that continuing to push for a low-cost reliable and widely available quantitative test should be our goal.  In the meantime, the FST fills an important gap.  I think these points are important to make in the paper.

 One other very minor suggestion that would help clarify 2018 terminology would be to say  “Early recognition of G6PD deficiency in newborns, prompting the implantation….can help prevent *acute bilirubin encephalopathy and thus kernicterus/kernicterus spectrum disorder*." ^1^

We have read this submission. We believe that we have an appropriate level of expertise to confirm that it is of an acceptable scientific standard.

## References

[1] Le PichonJBRiordanSMWatchkoJShapiroSM: The Neurological Sequelae of Neonatal Hyperbilirubinemia: Definitions, Diagnosis and Treatment of the Kernicterus Spectrum Disorders (KSDs). *Curr Pediatr Rev*.2017;13(3) : 10.2174/1573396313666170815100214 199-209 10.2174/1573396313666170815100214 28814249

# Peer review: Referee response for version 1

**DOI:** 10.21956/wellcomeopenres.14520.r30556

**Published:** 2018-02-26

**Authors:** Ari W Satyagraha

**Affiliations:** 1 Eijkman Institute of Molecular Biology, Jakarta, Indonesia

This is a good study that is currently needed to inform whether G6PD FST test can be used to screen for G6PD deficiency in newborns. There are not many tests available for screening G6PD in newborns, especially quantitative tests that can be used in remote areas where proper laboratories are not available and hence rely on qualitative ones. Since the test used in this study is qualitative, it may missed the intermediate G6PD activities. As Mahidol is the most dominant G6PD variant in the region, it may give a bit of an interpretation problem because it usually present itself in the intermediate G6PD activities. 

 As the authors have discussed, quantitative tests may be used to screen for G6PD in newborns but the tests' cut offs have to be adjusted for newborns since they have higher reticulocytes (thus higher G6PD activities) than adults. 

 However, I have difficulties in finding the numbers of deficient males (84/786) and deficient females (9/735) from. I could not clearly find it from Table 1 or Figure 1. 

 In general, I found this study interesting and very helpful and the number of subjects screened were statistically sound.

I have read this submission. I believe that I have an appropriate level of expertise to confirm that it is of an acceptable scientific standard.

# Response

**Published:** 2018-03-12

**Authors:** Laurence Thielemans

**Affiliations:** 1Shoklo Malaria Research Unit, Mahidol-Oxford Tropical Medicine Research Unit, Faculty of Tropical Medicine, Mahidol University, Mae Sot, Thailand

We appreciate the comments of the reviewer and we agree with her that showing the results according to gender is usually preferred for G6PD data. In this case the focus of the analysis was the overall quality of the test in identifying phenotypic deficiency; therefore for simplicity we had pooled the results of males and females in Table 1. In particular, among the 85 deficient subjects identified in the clinics and confirmed deficient by QC, 78 were males and 7 were females. Among the1428 subjects identified as normal in the clinics and in the QC, 701 were males and 725 were females. For the discrepant results between the clinics and the laboratory QC, the sex and genotypes were already specified in the footnote of Table1.

# Peer review: Referee response for version 1

**DOI:** 10.21956/wellcomeopenres.14520.r29386

**Published:** 2018-01-15

**Authors:** Issarang Nuchprayoon

**Affiliations:** 1 Faculty of Medicine, Chulalongkorn University, Bangkok, Thailand

This work addresses an overlooked issue of underdiagnosis for G6PD deficiency when cord blood are used for G6PD testing. The methodology is sound and number of subjects were adequate. 

 The underdiagnosis may occur with qualitative test such as FST, as quantitative test will adjust for hemoglobin concentration to yield IU/g Hb. In the discussion, the author may consider a possibility that FST may underdetect deficiency in cord blood as hemoglobin concentration in cord, 15.7 +/- 1.1 g/dL [1], is much higher than one-month old babies, 11.7 +/- 1.6 g/dL [2]. The 25% higher red cell mass and G6PD activity could affect qualitative assessment of fluorescence in deficient samples.

 Another point that shoud be mentioned is that most G6PD deficient neonates of Karen origins are G6PD Mahidol, a mildly deficient variant. Some males with residual but deficient G6PD activity may fluoresced bright enough in FST and called normal at higher hemoglobin concentration such as cord blood. This phenomenon might not occur in severely deficient variant such as G6PD Viangchan.

I have read this submission. I believe that I have an appropriate level of expertise to confirm that it is of an acceptable scientific standard.

## References

[1] OzolekJ: Cord Blood Hemoglobin Screening: Normal Values, Sex Differences, Is it Necessary? 1314. *Pediatric Research*.1998;43: 10.1203/00006450-199804001-01335 10.1203/00006450-199804001-01335

[2] de Fátima da Silva Vieira MarquesRAugusto de Aguiar Carrazedo TaddeiJAncona LopezFAparecida Pellegrini BragaJ: Hemoglobin Values in the First Six Months of Life in Exclusively Breastfed Children. *Open Journal of Blood Diseases*.2013;03(04) : 10.4236/ojbd.2013.34024 130-135 10.4236/ojbd.2013.34024

# Response

**Published:** 2018-03-12

**Authors:** Laurence Thielemans

**Affiliations:** 1Shoklo Malaria Research Unit, Mahidol-Oxford Tropical Medicine Research Unit, Faculty of Tropical Medicine, Mahidol University, Mae Sot, Thailand

We appreciate the comments of the reviewer. We don’t really know how higher hemoglobin concentrations influence the reading of the FST, we did observe in the past that subjects with anemia tend to have a brighter FST when compared with subjects with normal hemoglobin concentration (Bancone et al., 2015). Nonetheless, this aspect certainly deserves more attention and we hope to investigate it further in the future. As for the Mahidol variant, we have never observed adult males with intermediate activity in the Karen population; depending on the reagents used for the gold standard spectrophotometric assay, the median enzymatic activity in Mahidol hemizygous males was well below 10% of the population median (using the WHO protocol with home-made reagents and after removal of WBCs from blood sample, Bancone et al., 2014) or 12.8% of the population median (using commercial kits on whole blood samples, Bancone et al., 2016). In the Karen population the allelic frequency of Viangchan mutation is very low so we have never been able to analyze an appropriate number of Viangchan hemizygous males to assess the enzymatic activity.
